# Double-Focusing Gradient-Index Lens with Elastic Bragg Mirror for Highly Efficient Energy Harvesting

**DOI:** 10.3390/nano12061019

**Published:** 2022-03-21

**Authors:** Jeonghoon Park, Geon Lee, Dongwoo Lee, Miso Kim, Junsuk Rho

**Affiliations:** 1 Department of Mechanical Engineering, Pohang University of Science and Technology (POSTECH), Pohang 37673, Korea; jh2park@postech.ac.kr (J.P.); leegeon96@postech.ac.kr (G.L.); dwlee93@postech.ac.kr (D.L.); 2 School of Advanced Materials Science and Engineering, Sungkyunkwan University (SKKU), Suwon 16419, Korea; smilekim@skku.edu; 3 Department of Chemical Engineering, Pohang University of Science and Technology (POSTECH), Pohang 37673, Korea; 4 POSCO-POSTECH-RIST Convergence Research Center for Flat Optics and Metaphotonics, Pohang 37673, Korea

**Keywords:** gradient-index lens, elastic Bragg mirror, energy harvesting, piezoelectric energy harvester, double-focusing platform

## Abstract

The applicability of piezoelectric energy harvesting is increasingly investigated in the field of renewable energy. In improving harvester efficiency, manipulating elastic waves through a geometric configuration as well as upgrading harvester elements is important. Periodic structures, such as phononic crystals and metamaterials, are extensively employed to control elastic waves and enhance harvesting performance, particularly in terms of wave localization and focusing. In this study, we propose a double-focusing flexural energy harvesting platform consisting of a gradient-index lens and elastic Bragg mirror. Based on the design process, the frequency and time response of the harvesting platform are analyzed. The results indicate that the output voltage and power calculated at 1800 Ω are 7.9 and 62 times higher than those observed in the bare plate, respectively. Even when compared to the existing gradient-index system, they are 1.5 and 2.3 times higher, respectively. These findings can facilitate the usage of periodic structures as geometric stimuli to significantly enhance harvesting performance.

## 1. Introduction

The development of self-powered and wireless network devices has aggravated the problem of charging them. The disadvantages of using conventional batteries include battery replacement and disposal issues. Energy harvesting, which can provide sustainable and decarbonized energy, is being actively researched to address this. Energy harvesting is the method of converting diverse energy sources into electrical energy using transducers, such as piezoelectric materials, pyroelectric materials, and solar cells. Among them, piezoelectric energy harvesting, which converts vibration (elastic wave) into electrical energy, is extensively researched owing to its high efficiency [1,2]. Piezoelectric materials used in piezoelectric energy harvesting can transform mechanical motion into electrical energy. Substantial polarization induced by the mechanical stress/strain applied to the piezoelectric material can improve piezoelectric energy harvesting efficiency. Therefore, wave focusing or localization through tailoring geometric designs of phononic crystals and metamaterials is essential for promoting highly efficient energy harvesting performance [3,4,5].

Recently, phononic crystals or metamaterials have been investigated with remarkable effectiveness to manipulate elastic waves [6,7,8]. These periodic structures can aid in effectively capturing elastic waves using piezoelectric energy harvesters. Additionally, existing barriers can be improved by harnessing the unique properties of wave–matter interaction at the wavelength scale. Local resonance, defect modes, and wave focusing are the three representative mechanisms for manipulating elastic waves with periodic configurations. Several resonance-based systems have been developed to localize the elastic wave using local resonance and defect modes of the structure [9,10,11,12]; however, they are unreliable for practical usage owing to their narrow frequency range. To resolve these rigorous restrictions, phononic crystals were employed in experiments based on lens-focused mechanisms. Tol et al. [13] pioneered the idea of using lens-type phononic crystals for energy harvesting. Piezoelectric energy harvesting of the Lamb wave was achieved using a gradient-index (GRIN) lens configured to exhibit a gradient refractive index by adjusting the inclusion of a unit cell. Zareei et al. [14] constructed a lens-like structure by modulating the plate thickness without changing the inclusion. Furthermore, Hyun et al. [15] proposed a GRIN lens harvester with control over the hole radius. Moreover, Tol et al. [16] improved the efficiency by focusing on both unidirectional and omnidirectional waves using a Luneburg lens.

Apart from the lens type, Carrara et al. [17] proposed the mirror-type wave focusing design. Herein, an elliptical-shaped structure was enabled by phononic crystals, and piezoelectric energy harvesting was accomplished using reflected waves. To improve the efficiency of energy harvesting and broaden the operating frequency range, Tol et al. [18] harvested energy using embedded beads on a plate. Subsequently, Darabi et al. [19] used multiple scattering formulations to develop a theoretical model of an elastic mirror composed of phononic crystals. Recently, Qi et al. demonstrated piezoelectric energy harvesting by focusing the wave on the desired position using a labyrinthine design [20]. However, no further breakthroughs or efficiency improvements have been reported by combining standard GRIN lenses with elastic wave mirrors.

In this study, we propose a novel and highly efficient energy harvesting platform that simultaneously incorporates elastic GRIN lens and elastic Bragg mirror (EBM). Herein, the elastic wave confined by the GRIN lens is scattered across the focal point, and the scattered wave is reflected off a phononic crystal mirror, thus achieving a double-focusing mechanism. We believe that the proposed harvesting platform forms a basis for the development of highly efficient and robust harvesting platforms that do not rely on inclusions.

The remainder of the paper is organized as follows. In Section 2, we describe the platform’s mechanism and the entire design process. Section 3 discusses the results of frequency and time analyses performed to evaluate the focusing and harvesting capabilities of the proposed platform. Finally, the study findings are summarized in Section 4. We expect that this harvesting platform paves the way for developing a highly efficient robust harvesting platform that does not rely on inclusions.

## 2. Design Process and Methods

**Figure 1:**
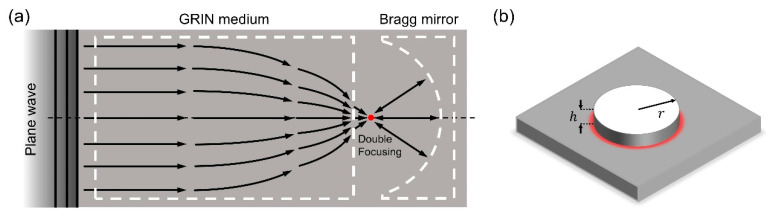
Schematic of the harvesting meta-platform. (**a**) Double-focusing mechanism with gradient-index (GRIN) medium and the semicircular elastic Bragg mirror (EBM); (**b**) Cylindrical piezoelectric energy harvester attached on the focal point.

We propose a considerably enhanced system, namely the GRIN-EBM plate, that captures flexural energy more effectively than a single GRIN lens plate that has been previously reported [15]. Herein, the GRIN lens and semicircular EBM mechanisms are integrated using phononic crystals. The GRIN-EBM plate is designed to focus the incident plane wave initially and then reflect the wave propagating away from the focal point, resulting in double-focusing at 30 kHz target frequency (Figure 1a). The mechanism of the proposed GRIN-EBM can be summarized as follows: (1) GRIN lens with gradually varying refractive index of each line of arrays bend the incident plane waves and focus them on a particular focal point [21]. (2) The focal point takes control of this newly generated point source. (3) As the semicircular phononic crystals generate a semicircular EBM, the wave is reflected and refocused on the same focal point. The wave focusing capability can be significantly increased in comparison with that of the existing system by continuing to focus the wave twice on the focal point [15]. Furthermore, the energy harvesting performance can be improved by attaching a piezoelectric device at the focal point in the direction perpendicular to the plate (Figure 1b).

**Figure 2:**
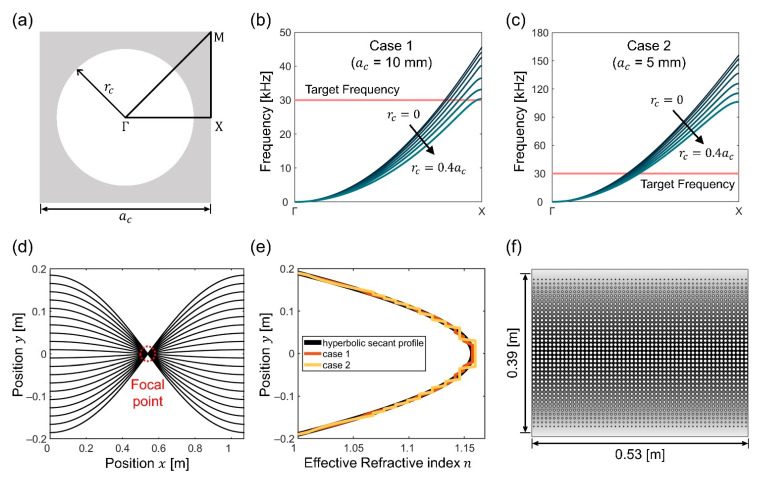
(**a**) Schematic of a square unit cell with a circular hole and the first irreducible Brillouin zone (Γ-X-M-Γ). Dispersion curves of considering the first flexural mode from $r_{c}=0$ to $r_{c}=0.4a_{c}$ for (**b**) case 1 and (**c**) case 2; (**d**) Ray-tracing of the field with continuous hyperbolic secant profile (black line in (**e**)); (**e**) Two-dimensional (2D) plot comparing the continuous hyperbolic secant index profile and discrete index profiles; (**f**) Schematic of the designed gradient-index (GRIN) plate.

The entire GRIN-EBM plate is designed in the order of GRIN plate, EBM plate, and GRIN-EBM plate. Initially, the refractive index of the unit cell of the GRIN lens should be calculated to design the GRIN plate. We consider a square unit cell with a circular hole as the unit cell for the GRIN lens; $a_{c}$ and $r_{c}$ denote the lattice constant and radius of the circular hole, respectively (Figure 2a).

A two-dimensional (2D) model rather than a three-dimensional model is considered using the Mindlin–Reissner plate theory [22,23,24], which enables the shear deformation of a plate to compute the dispersion relation of the GRIN unit cells with varying radii (Appendix A). We used 2-mm thick aluminum for the bare plate; herein, Young’s modulus E= 70 GPa, Poisson ratio v= 0.33, and mass density ρ= 2700 kg/m^3^.

Figure 2b (case 1: $a_{c}=10$ mm) and Figure 2c (case 2: $a_{c}=$ 5 mm) illustrate the computed dispersion relations. As indicated in the figures, the phase velocity ($v_{p}=2\pi f/k_{\Gamma x}$) of the dispersion curve decreases with the increase in the radii of the holes. Moreover, the phase velocity is inversely proportional to the effective refractive index ($n=v_{0}/v_{p})$, where $v_{0}$ denotes the elastic wave velocity of the propagation mode in the background medium. It is worth noting that when the range of $r_{c}$ exceeded 0.4$a_{c}$, the structure’s side becomes extremely thin, resulting in undesirable dynamic behavior. Furthermore, the group velocity varies sharply close to the end of the first Brillouin zone. As this may impede the performance of the GRIN lens, the maximum radius is set to $0.4a_{c}$, whereas the minimum radius is 0 mm.

Subsequently, we used the linear interpolation method with $\Delta k_{\Gamma X}=\left(\pi /a\right)/1000$ and assigned the minimum refractive index $n_{min}\left(r_{c}=0\right)$ to 1. The calculated maximum refractive index $n_{max}\left(r_{c}=0.4a_{c}\right)$ is 1.1572. The hyperbolic secant refractive index profile can be defined as [21]

(1) $$n\left(y\right)=n_{max}\mathrm{sech}\left(\alpha y\right),\;$$

where the gradient coefficient α can be calculated as

(2) $$\alpha =\frac{{\mathrm{sech}}^{-1}\left(\frac{n\left(y_{max}\right)}{n_{max}}\right)}{y_{max}}.$$

Furthermore, the focal length of the GRIN lens can be determined using Equation (3).

(3) $$f=\frac{\pi }{2\alpha }.$$

Based on the size of the entire system, we considered the maximum absolute value of the y-axis position $y_{max}$ as 0.19 m. Substituting $n_{max}$ and $y_{max}$ in Equation (2), we obtained α= 2.9138 m^−1^. Consequently, the focal length f= 0.539 m is obtained by substituting α in Equation (3). The numerical focal length and ray trajectories (Figure 2d) concur well when compared to the ray-tracing approach based on the hyperbolic secant refractive index profile.

The size of the unit cell determines the number of index profiles that can be represented in a specified amount of space. As case 1 matches the continuous hyperbolic secant profile more closely than case 2 (Figure 2e), the unit cell of the GRIN lens is set to $a_{c}=10$ mm. When the unit cell size exceeds 10 mm, the operating frequency of the GRIN lens reduces below 30 kHz because the first band of the flexural modes terminates at this frequency.

As the lattice constant of the GRIN unit cell is 10 mm, the wave focused by the GRIN lens on the focal point diverges further if the x-axis involves more than 53.9 arrays. Therefore, the number of x-axis arrays on the GRIN lens should be less than 53.9. We employed 53 arrays across the x-axis to fit the ray-tracing results in a continuous GRIN medium as closely as possible. As $y_{max}$ is set to 0.19 m in this study, 19 unit cells are distributed, and 39 unit cells are arrayed along the y-axis.

We set the radius interval at 0.1 mm to account for the manufacturing tolerance of laser sintering, which is essential for potential applications. Moreover, the radius with the refractive index closest to that of the hyperbolic secant profile (n(y)) is selected carefully (Table 1). Figure 2f illustrates the schematic of the designed GRIN plate.

**Figure 3:**
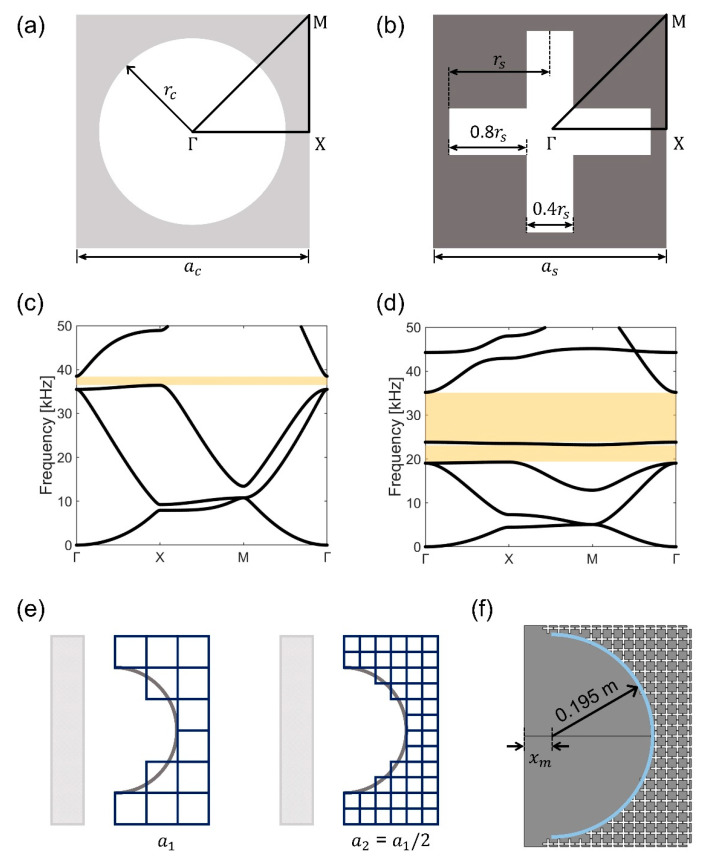
Schematic of the unit cell with a (**a**) circular hole and (**b**) cross-shaped hole. (**c**) Dispersion curves of the unit cell depicted in (**a**) with $a_{c}=20$ mm; (**d**) Dispersion curves of the unit cell depicted in (**b**) with $a_{s}=20\;$ mm. The yellow shaded area indicates the bandgap region; (**e**) Schematic of the elastic Bragg mirror (EBM) with different lattice parameters ($a_{1}$ and $a_{2}$ ); (**f**) Schematic of the EBM plate with $a_{s}=20$ mm.

After constructing the GRIN plate, we designed the EBM plate. The EBM is used to establish a double-focusing effect by reflecting the waves from the focal point of the GRIN lens and refocusing them as they exit the focal point. When a wave starts to propagate from one spot and is reflected by the semicircular mirror, it rebounds to its origin. We leveraged the Bragg bandgap of phononic crystals to reflect the wave without passing it through the bandgap. Unlike using a rigid wall mirror, the EBM has the benefits of being able to pass waves depending on the frequency and not damaging the overall plate structure. One of the most significant aspects in designing a semicircular EBM is representing the curvature of the circle, which requires a small lattice parameter ($a_{s}$) of the unit cell. Hence, we use a unit cell with a circular hole (Figure 3a) for the GRIN lens plate and a unit cell with a cross-shaped hole (Figure 3b) for the EBM plate. We set $a_{c}=a_{s}$ and $r_{c}=r_{s}$ for comparison. We observe that no bandgap exists around the target frequency of 30 kHz in the dispersion curve (Figure 3c) of the 20 mm × 20 mm × 2 mm unit cell with a circular hole; however, the bandgap appears in a relatively narrow range of approximately 37 kHz. This indicates that a larger unit cell is required to obtain a bandgap of 30 kHz. Conversely, the dispersion curve of the 20 mm × 20 mm × 2 mm unit cell with a cross-shaped hole (Figure 3d) generates an extremely broad bandgap around the target frequency of 30 kHz while maintaining its unit cell size; this implies that it can be an excellent candidate for the EBM plate. As indicated in Figure 3e, a smaller unit cell embodies the curvature of the semicircle more accurately than a larger unit cell. Therefore, the EBM unit cell in this study is 20 mm × 20 mm × 2 mm in size with a cross-shaped hole.

After setting the shape and size of the EBM unit cell, we drew a circle with a diameter of 0.39 m, the *y*-axis length of the GRIN lens. The EBM unit cell is arranged such that the area of the circle does not exceed 50% of the unit cell. Figure 3f illustrates the EBM prototype with an interval $x_{m}$ from the end line of the GRIN lens. We determined the value of $x_{m}$ after performing the frequency analysis on the GRIN-EBM plate while simultaneously evaluating whether the focal length changes.

## 3. Results and Discussion

**Figure 4:**
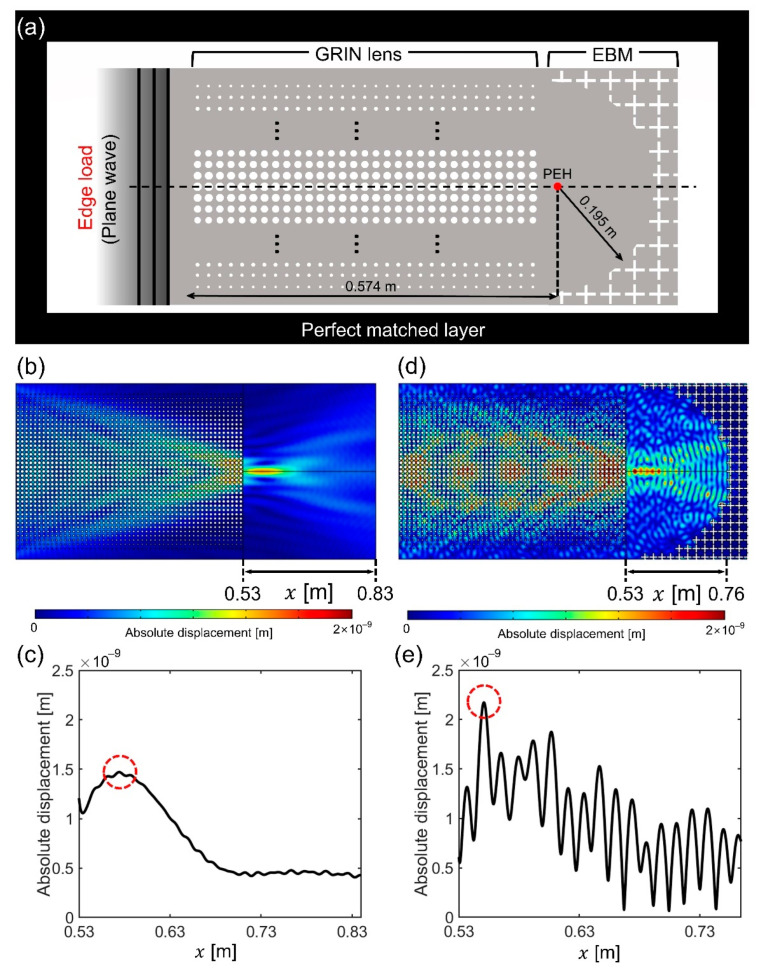
Frequency analysis of the gradient-index (GRIN) phononic crystals (PC) and GRIN-elastic Bragg mirror (EBM) plate. (**a**) Schematic of the frequency response simulation; (**b**) Displacement field of the GRIN plate; (**c**) Absolute displacement considering the *x*-axis position graph of the GRIN plate; (**d**) Displacement field of the GRIN-EBM plate; (**e**) Absolute displacement considering the *x*-axis position graph of the GRIN-EBM plate.

We performed a frequency response analysis using the commercial software COMSOL Multiphysics^TM^ version 5.6 to examine the wave focusing capabilities of the designed GRIN and GRIN-EBM plates. We imposed the perfect matched layer (PML) boundary condition to the plate surrounding the GRIN or GRIN-EBM plate to prevent unnecessary reflection at the boundary. Subsequently, we stimulated an input plane wave by applying the edge load per unit length (1 N/m) on the left side of the plate (Figure 4a). To clearly show the difference in wave focusing ability between the GRIN and GRIN-EBM plates, material loss is not considered in this study. Figure 4b,c show the results of the frequency response analysis of the designed GRIN plate. As the GRIN plate is not optimal in terms of a discrete hyperbolic secant profile with a small amount of impedance mismatch, the focal point is relocated to 0.574 m, which exhibits the highest absolute value of the displacement (Figure 4c). Therefore, the center of the semicircular EBM is set to 0.574 m.

The frequency responses of the designed GRIN-EBM plate with unit cell sizes of 10 mm (GRIN) and 20 mm (EBM) were found to be similar to that of the GRIN plate. Figure 4d depicts the absolute value of the flexural displacement field. The absolute displacement oscillates as a byproduct of the wave reflected from the EBM (Figure 4e). The high and low values indicate the quantities of constructive and destructive interferences, respectively. We also observed that the absolute displacement fields exhibit the highest value at x = 0.55 m (Figure 4e), which is 1.48 times larger than that of the GRIN plate (Figure 4c). The focal point of the GRIN-EBM plate is set to x = 0.55 m. Herein, we observe that the absolute value of the GRIN-EBM plate is not precisely twice that of the GRIN plate, and the focal length (0.55 m) differs from the center of the EBM (0.574 m). This can be attributed to two reasons: (1) As the GRIN plate is not optimal, the focal point is slightly broad and not a point. (2) Owing to its discrete curvature, EBM is not a perfect semicircular mirror. Nevertheless, the GRIN-EBM plate can capture more energy than the GRIN plate or the bare plate owing to its superior wave collection capacity.

**Figure 5:**
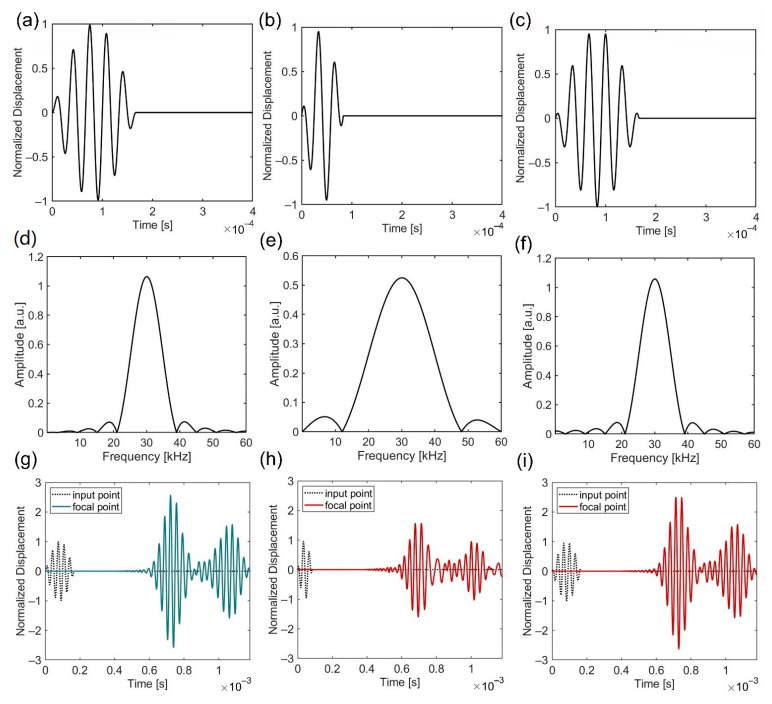
Time-harmonic analysis of the gradient-index and elastic Bragg mirror (GRIN-EBM) plate. (**a**) One cycle of the tone burst sine wave with m = 5; (**b**) One cycle of the modulated sine wave with $f_{m}$ = 6 kHz; (**c**) One cycle of the modulated sine wave with $f_{m}$ = 3 kHz; (**d**–**f**) represents the fast Fourier transform results of (**a**–**c**), respectively; (**g**) Two-dimensional (2D) displacement–time graph with (**a**) as an input wave; (**h**) 2D displacement–time graph with (**b**) as an input wave; (**i**) 2D displacement–time graph with (**c**) as an input wave. The displacement is normalized to the maximum amplitude of the input signal $w_{0}$.

Based on our analysis, we propose another significant function of the GRIN-EBM plate as follows. Although the duration of the input wave is insufficient to establish constructive interference and improve energy harvesting performance, the flexural wave energy is harvested twice (input and reflected waves), primarily owing to the double-focusing mechanism of the GRIN-EBM plate. Figure 5 illustrates the time-domain simulation of the GRIN-EBM plate to validate the double-focusing effect. We employ three types of input waves, including one tone burst sine wave and two modulated sine waves. The m-cycle input of the tone burst sine wave can be defined as

(4) $$w=w_{0}\sin\left(2\pi f_{m}t\right)\sin\left(\frac{2\pi f_{m}t}{m}\right),$$

where $w_{0}$ denotes the amplitude of the signal, and $f_{m}$ indicates the input wave frequency (30 kHz). The modulated sine wave can be obtained as

(5) $$w=w_{0}\sin\left(2\pi f_{m}t\right)\cos\left(2\pi f_{c}t\right)$$

where $f_{c}$ denotes the carrier frequency. The three different input waves considered are (1) a tone burst sine wave with m= 5 (Figure 5a); (2) a modulated sine wave with $f_{m}=$ 6 kHz (Figure 5b); and (3) a modulated sine wave with $f_{m}$ = 3 kHz (Figure 5c). When the frequency spectra of the three input waves (Figure 5d–f) are evaluated, the tone burst sine wave with m= 5 and the modulated sine wave with $f_{m}$ = 3 kHz exhibits comparable spectra; their 30 kHz component is 1.06 a.u. Conversely, the modulated sine wave with $f_{m}$ = 6 kHz exhibits relatively different spectra, wherein its 30 kHz component is 0.53 a.u. We conclude that the time-domain simulation results can reveal strong wave focusing capabilities in a rather broad frequency range. This is because unlike resonant systems with local resonators or defect modes, the GRIN lens and Bragg gap based on non-resonant structures do not rely significantly on a single frequency. Furthermore, the time-harmonic responses of the tone burst sine wave with m= 5 and the modulated sine wave with $f_{m}$ = 3 kHz are nearly identical, whereas the modulated sine wave with $f_{m}$ = 6 kHz exhibits the poorest focusing capability. Figure 5g–i depict the results of the time-domain analysis. The input wave is a prescribed displacement of the left edge of the plate, and the output displacement is calculated at the focal point of the EBM (0.55 m) and the input point. As indicated in the figure, the waves are focused twice in each case. Figure 5g,i illustrate the maximum displacements of 2.495$w_{0}$ and 2.568$w_{0}$, respectively, in the first focal time. Figure 5h depicts a maximum displacement of 1.566$w_{0}$, which is relatively lower than that observed in Figure 5g,i. Similarly, Figure 5g,i illustrate a maximum of 1.578$w_{0}$ and 1.574$w_{0}$, respectively, in the second focal time, whereas Figure 5h exhibits a maximum of 0.946$w_{0}$. Based on the comparison of Figure 5h,i, it is apparent that better focusing is achieved when more 30 kHz components are present. We conclude that this system operates at a broader frequency range than the resonance-based harvesting platform. This can be attributed to the wave focusing performance not degrading substantially, although the 30 kHz component is relatively small in Figure 5h.

To verify the energy harvesting performance of the GRIN-EBM plate, we used COMSOL Multiphysics^TM^ version 5.6 to perform the frequency-domain analysis with the boundary and initial conditions identical to those used in the frequency response analysis. Solid mechanics, electrostatics, and electrical circuit modules are applied for the coupling of mechanical and electrostatic fields. The cylindrical piezoelectric patch has two output electrodes embedded within it, a ground electrode on the upper surface of the piezoelectric patch, and a terminal electrode on the lower surface of the patch in contact with the surface of the GRIN-EBM plate. Additionally, a piezoelectric energy harvester (PEH) is attached to the maximum displacement point of the GRIN plate (0.574 m) and GRIN-EBM plate (0.55 m). A load resistor is used to obtain the output voltage and power from PEH, which is integrated into an electric circuit. Lead Zirconate Titanate (PZT-5H) is used as a PEH material. Table 2 summarizes the detailed properties of PEH. Since electrodes are very thin compared to the plate and PEH, we neglect the geometric configurations of electrodes.

**Figure 6:**
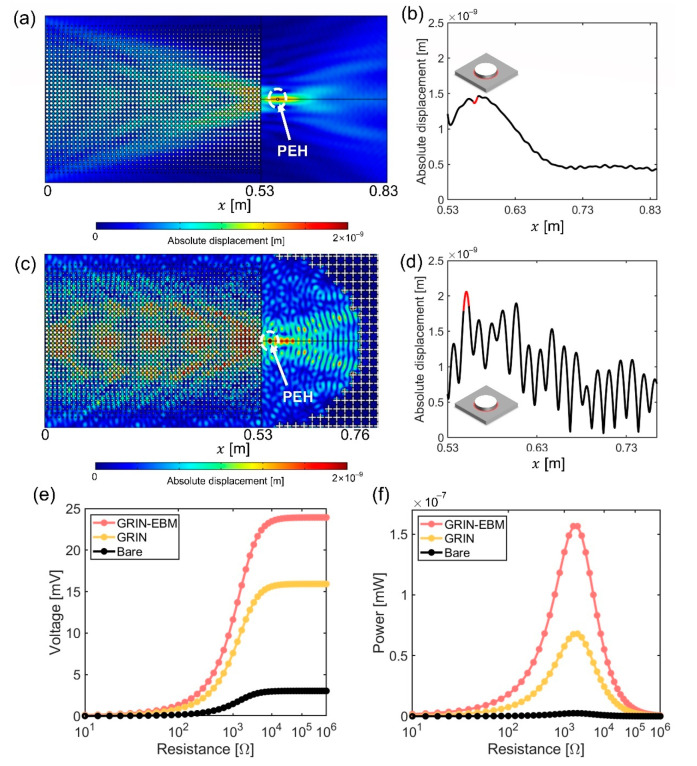
Frequency response analysis of the gradient-index (GRIN) and GRIN-elastic Bragg mirror (EBM) plate with a piezoelectric energy harvester (PEH). (**a**) Absolute value of the displacement field and (**b**) the two-dimensional (2D) centerline plot of the absolute value of the displacement per *x*-axis position of GRIN plate with PEH. The red line indicates the position of PEH; (**c**) Absolute value of the displacement field and (**d**) the 2D centerline plot of the absolute value of the displacement per *x*-axis position of GRIN-EBM plate with PEH. The red line indicates the position of PEH. 2D plots of the (**e**) voltage and (**f**) power, with respect to the load resistance.

Figure 6a,b illustrate the absolute values of the GRIN plate’s displacement field based on PEH and the displacement of the GRIN plate obtained at the centerline, respectively. Figure 6c illustrates the absolute value of the displacement field with PEH considering the GRIN-EBM plate, and Figure 6d illustrates the absolute value of the centerline displacement field. We observe that the displacement field changed slightly because the thickness of the harvester (0.2 mm) is considerably thinner than that of the bare plate (2 mm). To assess the energy harvesting performance of the harvesting platforms, the voltage is monitored while varying the load resistance from 10 Ω to 10^6^ Ω and connecting it to PEH using an electric circuit (Figure 6e). The voltage obtained at the GRIN-EBM plate at a load resistance of 1800 Ω is 1.5 and 7.9 times higher than that obtained at the GRIN and bare plates, respectively. The scale of the bare plate is the same as the GRIN-EBM plate. Figure 6f depicts the maximum power quantified based on the load resistance and voltage. The figure also illustrates the maximum power calculated when the load resistance is 1800 Ω. Moreover, the maximum power generated by the GRIN-EBM plate is 2.3 and 62 times higher than those of the GRIN and bare plates, respectively. The comparison with the existing GRIN system is more meaningful than the absolute voltage and power values because the input value is small (1 N/m). The study findings confirm the significant enhancement in wave focusing and energy harvesting performance in the proposed GRIN-EBM plate compared to those observed in the existing GRIN plate.

## 4. Conclusions

In this study, we developed a non-resonance-based GRIN-EBM plate, which serves as a highly efficient energy harvesting platform. Unlike previous studies that merely used either GRIN lenses or elastic mirrors, we incorporate both GRIN lens and semicircular EBM using repeated unit cells to establish the double-focusing mechanism. The unit cells and the entire system were designed in the order of GRIN and EBM, considering the target frequency and total system size. During the design process, the focal points of the GRIN and EBM were validated using frequency-domain analysis. The time-domain analysis indicated that the more target frequency components contained in the input signal, the better the performance of the GRIN-EBM plate. Furthermore, it also operates at a broader operating frequency range than resonance-based systems [12,25]. The frequency-domain analysis of the GRIN-EBM plate with PEH verified that the voltage and power generated by the GRIN-EBM plate are 1.5 and 2.3 times higher than the GRIN lens plate, respectively, under the same conditions. In general, the lattice constant of the GRIN and EBM unit cell should be increased in order to lower the operating frequency, but it can be compensated by implementing a new unit cell shape, reducing the number of GRIN arrays, and making the radius of the EBM smaller. We believe that our study findings form the basis for the development of double-focusing platforms, which are substantially more advanced than the standard single-focusing platform.

## Figures and Tables

**Table 1:** Position and radius of the gradient-index (GRIN) lens and corresponding refractive indices. Refractive indices are estimated to the nearest unit.

Position (y) [m]	Radius [mm]	$n\left(y\right)=n_{max}{\mathrm{sech}}^{-1}\left(\alpha y\right)$	$n_{eff}$
0.19	0	1	1
0.18	1.1	1.015	1.015
0.17	1.6	1.028	1.029
0.16	2	1.042	1.044
0.15	2.3	1.055	1.056
0.14	2.5	1.067	1.065
0.13	2.8	1.079	1.080
0.12	3	1.090	1.091
0.11	3.2	1.100	1.102
0.10	3.3	1.110	1.108
0.90	3.5	1.119	1.121
0.80	3.6	1.127	1.128
0.70	3.7	1.134	1.134
0.60	3.8	1.140	1.142
0.50	3.8	1.145	1.142
0.40	3.9	1.149	1.149
0.30	3.9	1.153	1.149
0.20	4	1.155	1.157
0.10	4	1.157	1.157
0	4	1.157	1.157

**Table 2:** Geometrical, mechanical, and electrical properties of a cylindrical piezoelectric energy harvester (Figure 1b).

Parameters	Value
Radius r	3 mm
Height h	0.2 mm
Density ρ	7500 kg/m^3^
$\mathrm{Elastic}\;\mathrm{constant}\;c_{11}$	127 GPa
$\mathrm{Elastic}\;\mathrm{constant}\;c_{12}$	80.2 GPa
$\mathrm{Elastic}\;\mathrm{constant}\;c_{13}$	84.7 GPa
$\mathrm{Elastic}\;\mathrm{constant}\;c_{22}$	127 GPa
$\mathrm{Elastic}\;\mathrm{constant}\;c_{23}$	84.7 GPa
$\mathrm{Elastic}\;\mathrm{constant}\;c_{33}$	117 GPa
$\mathrm{Elastic}\;\mathrm{constant}\;c_{44}$	23.0 GPa
$\mathrm{Elastic}\;\mathrm{constant}\;c_{55}$	23.0 GPa
$\mathrm{Elastic}\;\mathrm{constant}\;c_{66}$	23.5 GPa
$\mathrm{Piezoelectric}\;\mathrm{constant}\;e_{31}$	−6.62 C/m^2^
$\mathrm{Piezoelectric}\;\mathrm{constant}\;e_{31}$	−6.62 C/m^2^
$\mathrm{Piezoelectric}\;\mathrm{constant}\;e_{33}$	23.2 C/m^2^
$\mathrm{Piezoelectric}\;\mathrm{constant}\;e_{42}$	17.0 C/m^2^
$\mathrm{Piezoelectric}\;\mathrm{constant}\;e_{51}$	17.0 C/m^2^
$\mathrm{Relative}\;\mathrm{Dielectric}\;\mathrm{constant}\;\varepsilon_{11}$	1704
$\mathrm{Relative}\;\mathrm{Dielectric}\;\mathrm{constant}\;\varepsilon_{22}$	1704
$\mathrm{Relative}\;\mathrm{Dielectric}\;\mathrm{constant}\;\varepsilon_{33}$	1434

## Data Availability

The data presented in this study are available on request from the corresponding author.

## Appendix A

Considering the time-harmonic flexural displacement $u\left(x,t\right)=u_{0}e^{iwt}$, the governing equation of the flexural displacement of a Mindlin–Reissner plate can be determined as [22,24]

(A1) $$L^{T}DL\theta +G\left(\nabla w-\theta \right)=-\frac{1}{12}\rho h^{3}u^{2}\theta ,$$

(A2) $$\nabla^{T}\left(G\left(\nabla w-\theta \right)\right)=-\rho h\omega^{2}u,$$

where L denotes a differential operator, D indicates the flexural rigidity tensor, G represents the shear modulus tensor, h denotes the thickness of the plate, w indicates the flexural displacement, and θ represents the rotation vector. By imposing the Bloch-periodic condition ($u\left(x,t\right)=u_{0}e^{ik\cdot x},\theta \left(x,t\right)=\theta_{0}e^{ik\cdot x})$ on Equations (A1) and (A2), the governing equation can be discretized using the standard Galerkin method [22], as follows:

(A3) $$\left(K-\omega^{2}M\right)u=0,$$

where K denotes the reduced stiffness matrix and M indicates the reduced mass matrix. The solution of Equation (A3) with respect to the angular frequency ω for wave vector k ranging from 0 to π/a yields the dispersion relation of the GRIN lens unit cell.
